# Appropriate leadership in nursing home care: a narrative review

**DOI:** 10.1108/LHS-04-2020-0012

**Published:** 2021-03-24

**Authors:** Nick Zonneveld, Carina Pittens, Mirella Minkman

**Affiliations:** 1 Tilburg University, TIAS School for Business and Society, Tilburg, The Netherlands and Vilans, National Centre of Expertise in Long Term Care, Utrecht, The Netherlands; 2 Vrije Universiteit, Athena Institute, Amsterdam, The Netherlands; 3 Tilburg University, TIAS School for Business and Society, Tilburg, The Netherlands and Vilans, National Centre of Expertise in Long Term Care, Utrecht, The Netherlands

**Keywords:** Effects, Leadership, Nursing homes, Behavior, Factors

## Abstract

**Purpose:**

The purpose of this paper is to synthesize the existing evidence on leadership that best matches nursing home care, with a focus on behaviors, effects and influencing factors.

**Design/methodology/approach:**

A narrative review was performed in three steps: the establishment of scope, systematic search in five databases and assessment and analysis of the literature identified.

**Findings:**

A total of 44 articles were included in the review. The results of the study imply that a stronger focus on leadership behaviors related to the specific context rather than leadership styles could be of added value in nursing home care.

**Research limitations/implications:**

Only articles applicable to nursing home care were included. The definition of “nursing home care” may differ between countries. This study only focused on the academic literature. Future research should focus on strategies and methods for the translation of leadership into behavior in practice.

**Practical implications:**

A broader and more conceptual perspective on leadership in nursing homes – in which leadership is seen as an attribute of all employees and enacted in multiple layers of the organization – could support leadership practice.

**Originality/value:**

Leadership is considered an important element in the delivery of good quality nursing home care. This study provides insight into leadership behaviors and influencing contextual factors specifically in nursing homes.

## 1. Background

Leadership is seen as essential for the creation of cultural and structural change within organizations and the delivery of good quality nursing home care (Anderson *et al.*, 2005; Martin and Learmonth, 2012). Various studies confirm that leadership affects e.g. business management, information flows, health-related quality indicators, long-term vision, organizational structure, organizational culture, work environment and quality of care in nursing homes (Anderson *et al.*, 2005; Castle and Decker, 2011; Cummings *et al.*, 2010; Jeon *et al.*, 2015). Therefore, more insight is needed into how leadership should look to contribute to organizational and cultural change in nursing home care.

Leadership can be defined as “a process whereby individual influences a group of individuals to achieve a common goal” (Hunt, 2004, p. 3). Based on a review of leadership literature, Hunt (2004) distinguishes four common features of leadership. Leadership: is a process, involves influence, occurs in a group context and involves goal attainment. Leadership theory still divides leadership styles into two main groups: relationship-oriented leadership styles and task-oriented leadership styles. While relationship-oriented leadership focuses on individual persons and relationships, task-oriented leadership aims at the accomplishment of tasks. This division could also be interpreted as transformational leadership and transactional leadership (Avolio *et al.*, 1999). Transformational leadership is reflected in a process, in which a leader connects with his/her followers, with the aim of increasing intrinsic motivation to enhance performance. The driving force is a shared vision. Transactional leadership is a more top-down style, focusing on transactions between the leader and followers. There are clear structures, rules and procedures and the extrinsic motivation of employees is addressed (Avolio *et al.*, 1999). An example of transactional leadership is giving a personal reward for employees that achieve a certain goal, like a financial bonus. In our study, the two main streams of transformational and transactional leadership are used as an ordering framework, supplemented with a contingency approach category: context-dependent leadership styles. These styles assume that there is no universal leadership style and that different contexts and circumstances require different leadership styles (Northouse, 2018).

Various publications have been written about leadership in nursing home care. In most of these studies relational and transformative-related leadership styles are considered to be most appropriate in a nursing home and aged care (Anderson *et al.*, 2005; Corazzini *et al.*, 2015; Jeon *et al.*, 2015). The focus of most studies is the relationship between particular leadership styles and desired outcomes rather than understanding the behaviors and context behind them. However, as leadership is a process that takes place between people (Hunt, 2004), it consists of many components and influencing factors. It could also take place at multiple places in an organization, both formally and informally. The relationship between how leadership is executed and the outcomes achieved are, therefore, not simple or linear (Northouse, 2018). Therefore, more in-depth knowledge is required regarding leadership behaviors, the effects and the factors influencing them.

This study aims to provide a deeper understanding of what leadership is appropriate in nursing home care, also considering the changing context. To this end, various leadership behaviors, their effects and their influencing factors are examined by performing a narrative literature review with a systematic search.

## 2. Method

The objective of this study is to synthesize the existing evidence on leadership that best matches nursing home care, with a focus on behaviors, effects and influencing factors. A narrative review with a systematic search was conducted, drawing on the principles of hermeneutic review (Boell and Cecez-Kecmanovic, 2014). A hermeneutic review has two main characteristics:

1. accessing and interpreting the literature and
2. developing an argument.

The literature search is not only systematic but also flexible and iterative. As the identified literature increases, initial insights and ideas arise and less relevant literature could be rejected through progressive focus. It is argued elsewhere that a narrative review like a hermeneutic review should be the method of choice for interpreting a large and diverse set of literature in which authors have approached the topic differently (Greenhalgh *et al.*, 2018), as is the objective of our literature review. The review was executed in three steps: definition of scope, systematic search and assessment and analysis of the literature identified. To structure these three steps, a search protocol was developed beforehand.

### 2.1 Step 1: Establishment of scope

Definition of the search area included the formulation of a set of inclusion criteria. Manuscripts were included if they:

1. studied leadership;
2. targeted long-term care, nursing home/facility care or elderly care and were thereby applicable to the current nursing home care context;
3. addressed at least one of the main concepts of the study objectives, namely:
  - behavioral characteristics of leadership;
  - effects of leadership; and/or
  - factors influencing leadership;
4. were published between 2007 and December 2019 (because of the rapidly changing context);
5. were written in English; and
6. presented research findings of empirical work or reviews.

The search terms were developed through an iterative process in which three researchers were involved. Based on the two main elements of the study objectives – leadership and nursing home care – multiple search terms and combinations were explored in two databases (Pubmed/Medline and EBSCO).

### 2.2 Step 2: Systematic search

Using the terms described, systematic searches were performed in the PubMed/Medline, Cochrane, Cinahl, PsycInfo and Google Scholar databases. The snowballing technique was also applied: i.e. the reference lists of all articles included were studied to identify any additional relevant literature. After identifying all potentially relevant literature, assessment and analysis of the articles took place.

### 2.3 Step 3: Assessment and analysis of the literature identified

Assessment and analysis of the literature took place in three steps: the articles were screened based on the title, abstract and full-text to determine inclusion, data extraction took place and analysis was carried out on the articles that had been included.

1. Screening on the title, abstract and full-text: All titles and abstracts were screened independently by two researchers to decide whether articles met the inclusion criteria. If the two researchers assessed the article differently, a third researcher was consulted. The full-text of the selected articles was then independently assessed for eligibility by two researchers. Again, a third researcher was consulted if there was any disagreement. For this, the principles of the hermeneutic review were applied, meaning that the inclusion of articles in a later stage (for instance, full-text screening) was stricter due to progressive insights.
2. Data extraction: Two types of data were extracted from the articles. First, for each article the author(s), year of publication, journal, title, country, study design and applied methods, sector and organizational level were noted. Second, the main findings of the articles were extracted. The data extraction took place by two researchers, supervised by a third researcher. A fourth researcher was consulted if there was any disagreement.
3. Analysis: Descriptive analysis was chosen, as a large and diverse set of articles was included in which leadership was approached and studied differently. As the aim was to build an understanding of leadership, the analysis focused on interpreting the findings of the articles included. Reflection on the content analysis took place with a fourth researcher.

## 3. Results

**Figure 1.**
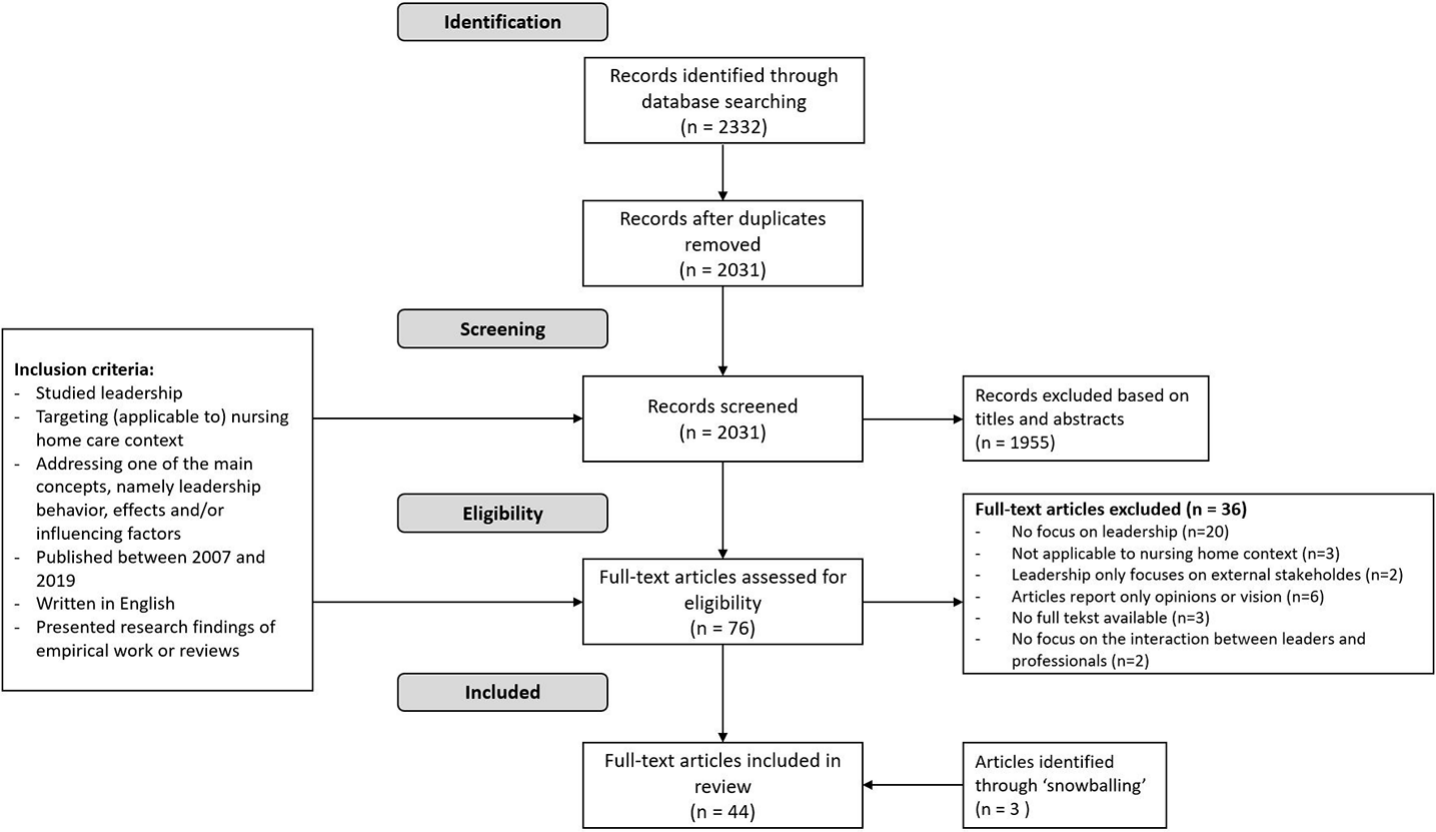
PRISMA flow chart

The systematic database search resulted in the identification of 2,332 scientific articles. After removal of duplicates, 2,031 records were screened on title and abstract, based on the formulated inclusion criteria. After this screening 76 scientific articles remained for full-text assessment. During the full-text screening, 36 papers were excluded due to the following reasons: no focus on leadership (*n *=* *20), not applicable to the nursing home care context (*n *=* *3), leadership only focuses on external stakeholders (*n *=* *2), articles report only opinions or vision (*n *=* *6), no full text available (*n = *3) and no focus on the interaction between leaders and professionals (*n *=* *2). As a result of the “snowballing” technique, 3 extra scientific articles were included. This resulted in a total of 44 included articles. Figure 1 shows the PRISMA flow chart, which displays the systematic literature search process. Table 1 presents the characteristics of the articles, including an overview of study design: 17 qualitative, 16 quantitative, 4 mixed methods and 7 (systematic) reviews were included.

### 3.1 Leadership behaviors

The articles included in this review describe many sorts of leadership behaviors. In most articles, the studied set of leadership behaviors is given a name/title/term such as “partnered leadership,” “connective leadership” and “adaptive leadership.” In the articles, they are often connected to one of the main leadership styles. Descriptions of leadership behaviors identified are, therefore, distributed over three main categories: relationship-oriented leadership, task-oriented leadership and context-dependent leadership.

Especially more recent articles move away from leadership styles and focus more on behaviors essential for new developments in nursing homes. In the articles of Brodtkorb *et al.* (2019) and Backman *et al.* (2018), important leadership characteristics were identified to support the implementation of person-centered care. Havig and Hollister (2018) focused on the interplay of independent workgroups (resembling self-organization) and appropriate supportive leadership.

#### 3.1.1 Relationship-oriented leadership behavior.

In total, 15 different sorts of leaderships related to relationship-oriented leadership were identified in 34 articles. Transformational leadership was studied the most (*n *=* *16), followed by relational leadership (*n *=* *7) and the resonant, coaching, consensus and consultative autocratic behavioral styles (*n *=* *3). Although “partnered leadership” (Jennings *et al.*, 2011; Leutz *et al.*, 2010), “individualized consideration” (Cummings *et al.*, 2010), “authentic leadership” (Hakanson *et al.*, 2014) and “connective leadership” (Jennings *et al.*, 2011) were also mentioned as research objectives, no outcomes regarding these behaviors were given in the articles.

When looking more closely at behaviors, the leadership types identified shows a lot of overlap. In relationship-oriented leadership behaviors identified, leaders focus on relationships, using emotional skills such as listening and empathy, to increase the involvement of employees (Cummings *et al.*, 2008; Forbes-Thompson *et al.*, 2007; Havig *et al.*, 2011; Havig *et al.*, 2011; Jeon *et al.*, 2015). As Havig *et al.* (2011) describe: “relationship-oriented style constitutes the behaviors of supporting […], developing […] and recognizing” (Havig *et al.*, 2011, p. 2). Transformational leadership aims to create awareness and involvement of employees in line with the objectives of the organization (Nielsen *et al.*, 2010).

#### 3.1.2 Task-oriented leadership behaviors.

Task-oriented leadership behaviors were studied less extensively. Furthermore, they were often contrasted with relationship-oriented leadership behaviors. In total 9 task-oriented types of leadership were mentioned as study objectives in 9 of the articles included in the review. In most of the articles, no specific characteristics were described.

The similarity between task-oriented and transactional leadership behavior is that it is rational, concise and business-like. Task-oriented leadership deals with the management of tasks and activities (who does what, when and how), distribution of roles, objectives, monitoring and results (Havig *et al.*, 2011; Havig *et al.*, 2011). Transactional leadership takes transactions between leaders and employees as a starting point (Cummings *et al.*, 2010). In terms of behavior, this is reflected in rewarding and “punishing” employees. “Shareholder management” is characterized by behavior in which minimal attention is paid to the development of relationships between people (Havig *et al.*, 2011). Employees work relatively autonomously, there is not much communication and decisions are made centrally. Autocratic leaders also make their own decision, but their employees face a relatively low amount of autonomy (Castle and Decker, 2011; Donoghue and Castle, 2009; Havig *et al.*, 2011).

#### 3.1.3 Context-dependent leadership behaviors.

Although the studies in this review focus predominantly on relationship-oriented leadership behaviors, the literature also recommends interpreting with caution. Various studies emphasize that leadership is a complex phenomenon that depends on situations and individuals (Jennings *et al.*, 2011). Some of the articles conclude that a combination of leadership behaviors is needed. Havig *et al.* (2011) conclude that a combination of both relationship-oriented and task-oriented leadership behaviors is preferred in their study of job satisfaction in nursing homes (Havig *et al.*, 2011). Nursing homes with a more hierarchical structure and more mutual interdependence could benefit from task-oriented leadership and vice versa. The authors conclude that leadership is context-dependent. Jennings *et al.* (2011) state:

The strongest statement that can be made based on empirical studies is that it is unwise to view transformational leadership as a preferred style, particularly when this style is assessed independently of other leadership styles and organizational variables (Jennings *et al.*, 2011, p. 15).

Some leadership behaviors identified in this review embrace this context-dependency and represent a combination of leadership behaviors. Lynch *et al.* (2011) describe the application of situational leadership to residential care. This is characterized by multiple behaviors of a leader, depending on the situation and the individual. Corazzini and colleagues focus on “adaptive leadership,” which makes a distinction between technical and adaptive challenges (Corazzini *et al.*, 2015; Corazzini and Anderson, 2013). In this context, technical challenges refer to issues that can be easily defined and solved with the appropriate expertise or resources. Adaptive challenges, on the other hand, require new and innovative solutions which may also require a change in values or attitudes. Issues often include both technical and adaptive challenges, in which different leadership behaviors are needed (Corazzini *et al.*, 2015; Corazzini and Anderson, 2013).

Both situational and adaptive leadership is built on the belief that appropriate leadership behaviors should be chosen based on situation and context (Corazzini and Anderson, 2013; Lynch *et al.*, 2011, 2018). Situational leaders exhibit leadership behavior, which fits with a particular situation and adapts this behavior accordingly to achieve results in a planned way. Central to adaptive leadership, which has roots in complexity theory, is the belief that there are no fixed solutions for complex issues. The behavior of adaptive leaders can, therefore, be characterized as highly flexible and adaptive, to cope with (sudden) changes and developments in complex environments (Table 2).

### 3.2 Reported effects of leadership

In 38 articles effects of leadership were described. The effects of leadership were measured quantitatively in 15 of the articles identified. In 4 articles effects were studied using mixed-methods, in 13 articles effects were measured using qualitative methods and effects were described in 6 reviews. The described effects in the qualitative articles are less “hard” and were not taken into consideration in the table. Reported effects can be separated into five categories: the effects of leadership on:

1. employees;
2. quality of care;
3. quality of life;
4. person-centered care; and
5. innovation processes.

Table 3 presents the effects studied in these articles.

Most studies report that relationship-oriented leadership has a positive impact on employees. Relationship-oriented leadership leads to higher job satisfaction (Cummings *et al.*, 2010; Donoghue and Castle, 2009; Havig *et al.*, 2011; Nielsen *et al.*, 2010), a better relationship with work (for example, a higher organizational commitment) (Cummings *et al.*, 2010; Donoghue and Castle, 2009; Lundgren *et al.*, 2016; Nielsen *et al.*, 2010), higher productivity and effectiveness (Buljac-Samardzic and van Woerkom, 2015; Cummings *et al.*, 2010) and more empowerment and development opportunities (Cummings *et al.*, 2014, 2010; Lundgren *et al.*, 2016; Nielsen *et al.*, 2008). Among the articles is one systematic review (Cummings *et al.*, 2010), in which 53 articles are studied. This study concludes that relationship-oriented leadership is more likely to have positive effects on employees.

In 11 of the articles, the relationship between leadership and quality of care was studied. In these articles, different effects were observed. In four articles no effects were found (Jeon *et al.*, 2015; Marotta, 2010; Olinger, 2010; Westerberg and Tafvelin, 2014). Four papers conclude that relationship-oriented leadership results in a higher quality of care (Castle and Decker, 2011; Harvath *et al.*, 2008; McKinney *et al.*, 2016; Westerberg and Tafvelin, 2014), while in one article it is concluded that a combination of task-oriented and relationship-oriented leadership leads to a higher quality of care (with the emphasis on task-oriented leadership) (Jennings *et al.*, 2011). Based on their study in Sweden, Westerberg and Tafvelin (2014) present an indirect positive relationship between transformational leadership and quality of care, via mediating variables such as organizational support, support by experienced colleagues, workload and control (Westerberg and Tafvelin, 2014). In all articles quality of care is either not defined consistently or not defined at all. One article studied the impact of leadership on quality of life in the USA. McKinney *et al.* (2016) report that consensus leadership behavior is “associated with a lower likelihood of deficiencies for quality of life” (McKinney *et al.*, 2016, p. 230).

Furthermore, in three articles a relationship between leadership and person-centered care is described (Backman *et al.*, 2016; Brodtkorb *et al.*, 2019; Lynch *et al.*, 2011). Backman *et al.* (2016) for instance conclude that there is a significant relationship between the leadership behavior (of older managers) and person-centered care and psychosocial climate. In this Swedish study, the most appropriate type of leadership and the associated behavior is not specified. Concerning the implementation of person-centered care, Backman *et al.* (2016) mention “Person-centered care moderates the relationship between leadership behavior” (Backman *et al.*, 2016, p. 8). The authors conclude that leadership is more important in organizations that offer less person-centered care. In these organizations, leaders need to provide direction toward a more person-centered way of working. In line with this, Brodtkorb *et al.* (2019) revealed: “a close connection between leadership style [participative leadership] and culture change processes toward PCC” (Brodtkorb *et al.*, 2019, p. 134).

On the other hand, a number of studies present contrasting findings or caveats (weak or even no evidence) with respect to the positive effects of relationship-oriented leadership (Harvath *et al.*, 2008; Havig *et al.*, 2011; Jennings *et al.*, 2011; Jeon *et al.*, 2015; Marotta, 2010; Olinger, 2010). In a Norwegian study, Havig *et al.* (2011) report that task-oriented leadership has a more significant impact on the job satisfaction of employees. Also, Jennings *et al.* (2011) conclude that there is little empirical evidence to relate impacts to certain leadership because leadership is multidimensional and complex: leaders use combinations of leadership behaviors and styles in practice. Olinger (2010) found no statistical significance for nursing home administrator and nursing director leadership styles on care quality.

### 3.3 Factors influencing leadership

Out of all articles included in this review, 22 articles describe factors that could influence leadership. The influencing factors were identified at three levels: the leader, the team(s) and the organization. Table 4 presents these factors.

A number of influencing factors – found in seven articles – can be related to the leader him/herself: personal characteristics of the leader (Cummings *et al.*, 2008, 2014; Nielsen and Cleal, 2011), leadership competencies (Cummings *et al.*, 2008), educational activities (Cummings *et al.*, 2008; Hakanson *et al.*, 2014; Vesterinen *et al.*, 2009) and distance to practice (Havig and Hollister, 2018; Kristiansen *et al.*, 2016). The systematic review by Cummings *et al.* (2008) provides particular insight into the influence of these factors on relational leadership. Cummings *et al.* state that the personal characteristics of effective leaders relate to openness, extraversion and management motivation. “Significant positive relationships were reported between the leaders’ motivation and their leadership behaviors.” (Cummings *et al.*, 2008, p. 244). Education of leaders, both in relation to professional knowledge and to leadership skills, is mentioned as a positive influencing factor in three articles (Cummings *et al.*, 2008; Hakanson *et al.*, 2014; Vesterinen *et al.*, 2009). In a Swedish case study, Hakanson *et al.* (2014) found that leaders identify their own shortcomings and needs for personal development by following educational activities. The specific content of the different educational activities or programs were not described in the articles. A distance to practice was found to be a constraining factor (Havig and Hollister, 2018; Kristiansen *et al.*, 2016). As illustrated by Havig and Hollister (2018):

They also spent less time at the ward and did not have the same knowledge about their employees’ work situation as the leaders in the high-quality wards. The result of this lack of leadership was often poor work environments, with interpersonal conflicts and frustration, which distracted the care workers and turned their focus away from their daily work duties and the residents (Havig and Hollister, 2018, p. 379).

Ten studies showed that team-related factors could influence leadership:

1. turnover and absence (Cloutier *et al.*, 2016; Havig *et al.*, 2011);
2. interpersonal relations (Corazzini *et al.*, 2015; Havig and Hollister, 2018);
3. workload (Corazzini *et al.*, 2015; Westerberg and Tafvelin, 2014);
4. willingness to be coached (Cummings *et al.*, 2014; Havig *et al.*, 2011);
5. employee well-being and satisfaction (Cummings *et al.*, 2014; Nielsen *et al.*, 2008);
6. self-efficacy (Nielsen *et al.*, 2009; Nielsen and Munir, 2009); and
7. interdependent workgroups (Havig and Hollister, 2018).

Two articles relate a high turnover and/or absence rate of employees to less effective leadership (Cloutier *et al.*, 2016; Havig *et al.*, 2011). In a Western Canadian case study, Cloutier *et al.* (2016) report that “With greater staff mobility and change, the leadership had less knowledge of their staff to mobilize existing skill sets, use the expertise and build cohesion” (Cloutier *et al.*, 2016, p. 12). Close interpersonal relations – staff/staff, leader/staff and staff/resident – were found to be positively related to leadership (Corazzini *et al.*, 2015; Havig and Hollister, 2018). In turn, a high workload was negatively related (Corazzini *et al.*, 2015; Westerberg and Tafvelin, 2014). Also, the (un)willingness of teams to be coached was mentioned as an influencing factor (Cummings *et al.*, 2014; Nielsen *et al.*, 2008). Cummings *et al.* illustrate this as follows:

“Some managers reported out that some of their staff have little interest in learning new things and updating their skills and knowledge,” as per the following quote: “They just want to do their job and go home.” […] A manager, who considered coaching uninterested staff to be undesirable, reported: “Not wanting to rock the boat (don’t have time to risk losing that staff)” (Cummings *et al.*, 2014, p. 205).

Furthermore, employee well-being and satisfaction were stated as potential influencers of leadership. Although there is limited evidence of the direct relationship between leadership behavior and well-being (Nielsen *et al.*, 2008), two articles mention that a higher level of job satisfaction corresponds to more effective leadership (Cummings *et al.*, 2014; Nielsen *et al.*, 2008). Finally, Havig and Hollister (2018) found that independent workgroups (or teams) of caregivers, which had their own meetings, reports and administrator, could have a possible influence on nursing home quality. Their analysis revealed that workgroups were fostered by three mediators, namely, psychological ownership, perceived insider status and shared mental models.

In total, 20 articles described factors that influence leadership at an organizational level. The following factors were identified in this category:

- organizational structure (Corazzini *et al.*, 2015; Cummings *et al.*, 2008, 2014; Lundgren *et al.*, 2016; Rokstad *et al.*, 2015);
- the extent to which person-centered care has been implemented (Backman *et al.*, 2016, 2020);
- organizational culture (Ali and Terry, 2017; Backman *et al.*, 2020; Corazzini *et al.*, 2015; Havig and Hollister, 2018; Jeon *et al.*, 2010; Nielsen *et al.*, 2008; Vesterinen *et al.*, 2009);
- the available information and information flow (Forbes-Thompson *et al.*, 2007; Hakanson *et al.*, 2014; Jeon *et al.*, 2010; Vesterinen *et al.*, 2009);
- previous leaders (Vesterinen *et al.*, 2009);
- available budget and time (Ali and Terry, 2017; Cummings *et al.*, 2014; Hakanson *et al.*, 2014; Nielsen *et al.*, 2010; Rokstad *et al.*, 2015);
- tasks and responsibilities (Hakanson *et al.*, 2014; Jeon *et al.*, 2010; Kristiansen *et al.*, 2016; Nielsen *et al.*, 2008);
- the leadership team (Hakanson *et al.*, 2014; Vesterinen *et al.*, 2009);
- organizational dynamics and stability (Jeon *et al.*, 2010; Nielsen *et al.*, 2010; Nielsen and Cleal, 2011);
- support from superiors (Jeon *et al.*, 2010; Westerberg and Tafvelin, 2014); and
- openness to change and innovations (Brodtkorb *et al.*, 2019; Jeon *et al.*, 2010; Lynch *et al.*, 2011; Nielsen *et al.*, 2008).

First, the structure of an organization was found to influence the way in which leadership is performed. In bigger organizations, for instance, there is often more distance between managers and the work floor than in smaller organizations and this creates challenges to performing direct, relational leadership (Lundgren *et al.*, 2016; Rokstad *et al.*, 2015). As Lundgren *et al.* state:

Physical distance between leaders and subordinates reduces the opportunity for leaders to supervise, organize and optimize nursing assistants’ work situations, which may have negative effects in the field of home help services (Lundgren *et al.*, 2016, p. 51).

In a Finnish qualitative study, Vesterinen *et al.* (2009) report that organizational culture and information available for employees influence leadership:

The managers said that their leadership style was influenced by the flow of information in the organization. It was difficult to lead others toward a vision when there was a lack of information (Vesterinen *et al.*, 2009, p. 508).

Other influencing factors include tasks and responsibilities of leaders (Hakanson *et al.*, 2014; Kristiansen *et al.*, 2016) and available budget and time. Although they emphasize that leadership depends on situations and people, Nielsen and Cleal (2011) relate a stable organization (low staff turnover, financially stable, no reorganizations) positively to (transformational) leadership.

## 4. Discussion

As a result of analysis of the academic literature currently available, the findings of this study provide insight into leadership behaviors, their effects and factors influencing them. When looking into what kind of leadership is considered appropriate in the nursing home care context, also considering its current developments, our analysis does not provide an unambiguous answer. Our review shows that leadership in nursing home care is a complex and multidimensional undertaking, influenced by multiple internal and external factors. On the one hand, there is a tendency toward relationship-oriented and transformational leadership in particular. Our search identified 15 different sorts of leadership related to relational leadership with many reported positive effects on health-care professionals, quality of care, quality of life and person-centered care. However, a diversity of measures was used, with a variety in quality. Both quantitatively and qualitatively observed effects were considered. On the other hand, contrasting findings have also been reported, for example, both positive and negative effects on job satisfaction associated with task-oriented leadership (Cummings *et al.*, 2010; Havig *et al.*, 2011). Also, various studies emphasized that “good” leadership cannot be achieved by applying only one type of leadership behavior. Both relationship-oriented and task-oriented leadership have resulted in positive effects, as demonstrated by the evaluation of job satisfaction in nursing homes (Havig *et al.*, 2011). Furthermore, as a broad scope was used to comprehensively identify insights applicable in nursing home care, the studies compared in this review were carried out in different contexts (for example, nursing homes, long-term care, facility care, etc.) in different countries using different methodologies. For example, in the studies included in which a relationship between leadership and quality of care was reported, different definitions of quality of care were used and there was no differentiation between specific aspects of quality of care (Castle and Decker, 2011; Havig *et al.*, 2011; Marotta, 2010; McKinney *et al.*, 2016; Olinger, 2010; Westerberg and Tafvelin, 2014). Therefore, it is also difficult to interpret and compare the results of these studies. This makes it hard to draw any meaningful conclusions about the effects of certain leadership. Another complicating factor in the identification of appropriate leadership is that leadership is a product of multiple influencing factors. Our review identified 22 influencing factors at the individual, team and organizational levels. This shows that leadership in nursing home care is not only complex and multidimensional but may also be influenced by internal and external factors. As a consequence, when looking for appropriate leadership, the answer does not lie in one type of leadership.

This observation is also reflected in some of the articles included in the review. Although a relationship-oriented style was the basis for investigation in most of the studies analyzed, some of them report that certain contexts and situations demand more task-oriented behaviors. Furthermore, literature also shows that the combination of both styles may be appropriate. A balanced mix of leadership styles, for instance, a relationship-oriented focus combined with task-oriented behaviors, is also advocated in other sectors outside nursing home care. Mintzberg (2009), for example, cites the broad variety of leadership styles in the literature and emphasizes that the application of one style may lead to management that is not in balance (Mintzberg, 2009). Furthermore, in their study on leadership patterns and their effects on employee satisfaction and commitment, Gavan O’Shea *et al.* (2009) conclude that effective leaders use a combination of styles (Gavan O’Shea *et al.*, 2009). This was also the conclusion reached by Aarons (2006) specifically with respect to the mental health sector (Aarons, 2006).

While our analysis shows a tendency in favor of combinations of elements from different types of leadership to deal with different situations and contexts, many included studies explore relationships between relational and task-oriented leadership only in a bivariate way. As Cummings *et al.* (2010) conclude:

In our analyzes, we had simplified the pattern of two approaches to leadership styles and their impact on specific outcomes for nurses, the nursing environment and the nursing workforce. In reality, leadership practices, behaviors and styles and outcomes are not that clean-cut (Cummings *et al.*, 2010, p. 17).

This awareness demonstrates that a greater focus on leadership behaviors in relation to contextual factors rather than leadership styles could provide more valuable insight into appropriate leadership in nursing home care. In most of the literature reviewed, however, leadership behavior is not described or explained precisely. Fortunately, more recent literature is moving away from studying solely leadership styles and is focusing more on appropriate leadership behavior for new developments, like the implementation of Dementia Care Mapping and person-centered care (Backman *et al.*, 2020; Lynch *et al.*, 2018; Quasdorf and Bartholomeyczik, 2019).

Another point worthy of reflection is that the results of our study show a broad variety of leadership terms, styles and names and a large degree of overlap between their characteristics. This is especially the case in the field of relationship-oriented leadership. It is debatable whether these different definitions of leadership really encompass different behaviors or only use different terminology.

Considering that a focus on leadership behaviors could provide more insight into effective leadership in nursing home care, it is interesting to ask what leadership behaviors will be appropriate with respect to the current developments in nursing home care. First of all, the nursing home care sector could be considered as a complex adaptive system (CAS), in which the connected elements of the system evolve and adapt continuously (Meadows, 2008). The current developments, with tendencies toward decentralization, self-organization and person-centered care, are examples of this evolving and adapting system. While the nursing home care sector consists of many different entities and a high level of interactivity, nursing homes can also be considered as systems in which organizational dynamics take place (Ashmos *et al.*, 2000). The consequence of leadership behavior is that it is important to be aware that employees are part of a complex system, both in the organization and in the health system as a whole. As complexity scientists (Lichtenstein *et al.*, 2006) reflect: “leadership is a dynamic that transcends the capabilities of individuals alone; it is the product of interaction, tension and exchange rules governing changes in perceptions and understanding.” (Lichtenstein *et al.*, 2006, p. 2). In this complex environment, it is important to reflect continuously and analyze the suitability of leadership behaviors in different contexts and situations. Corazzini *et al.* (2015) elaborate on this in their study about adaptive leadership and they conclude that problems in nursing homes are mostly complex and cannot be solved by one type of leader.

Furthermore, current developments toward flat organizations, decentralization and self-direction, show a tendency toward more collective responsibility and ownership at all layers of organizations. Most papers included in this review addressed a specific organizational level. A number of studies focus on leadership in middle management (Buljac-Samardzic and van Woerkom, 2015; Corazzini and Anderson, 2013; Hakanson *et al.*, 2014; Leutz *et al.*, 2010; Nielsen *et al.*, 2010; Nielsen and Cleal, 2011; Oldenhof *et al.*, 2016; Vesterinen *et al.*, 2009) and only one article is specifically taking independent workgroups (teams) into account (Havig and Hollister, 2018). Other articles cover board/management level and some do not focus on a specific organizational level. In the light of current developments in nursing home care, taking new organizational structures with decentralized collective responsibilities such as self-directed teams, into account, a focus on leadership across multiple layers of nursing homes would provide more detailed insights into leadership behaviors and the complex interaction between people and situations. It is striking that the current review did only identify one article that focused specifically on these issues.

### 4.1 Research limitations and implications

The literature review was carried out in a structured and systematic way. Six systematic reviews were used in this study, which included 255 articles in total (including several studies published before January 1, 2007). This provided a strong theoretical basis, including insights into a broader context. Because the leadership literature is extensive, only articles applicable to nursing home care were included. On the one hand, the current tendencies and insights in leadership literature are well represented in the literature applicable to nursing home care. On the other hand, the leadership literature in this sector is still relatively new. This may yield articles that take an exploratory approach. Also, the definition of “nursing home care” may differ between countries as will the services or care which are captured under this term. Furthermore, relevant insights in nursing home care are often shared in non-academic documents or grey literature. This study only focused on the academic literature. This “publication delay” could explain that literature on relatively new leadership-related tendencies such as self-organization, self-management and autonomous teams, was not available.

The results of this study show that a broad range of leadership behaviors is evident in nursing home care. Further investigation of behaviors that match particular contexts or situations would be relevant. The behaviors identified in this review provide insight into leadership in nursing home care, but more research is needed on how this is reflected in practice. Characteristics such as involvement and appreciation mainly focus on the result of leadership behavior, while more knowledge could be gained about how to actually achieve this. Future research should focus on strategies and methods for the translation of leadership into behavior in practice. Another relevant avenue of research is the impact of cultural aspects on leadership. Research demonstrates that leadership-related culture and values may differ across settings and countries (Ardichvili and Kuchinke, 2002; Chhokar *et al.*, 2007; Hofstede, 2011). Examples are power distance, masculinity, uncertainty avoidance and long-term orientation (Hofstede, 2011). These core values could influence leadership approaches and behaviors in practice. Our review includes studies from various, mainly Western, countries such as the USA, Canada, Australia, England and multiple Scandinavian countries. The included articles do not explicitly reflect on the cultural aspects of leadership. More insight into what the exact impact of these aspects is would be relevant. Finally, an interesting research question would be to compare how leadership behavior is perceived by the different people involved. The role of informal leadership and the dynamics in collaborating networks could also be interesting topics for further research.

## 5. Conclusions

In conclusion, because leadership in nursing home care is multidimensional and influenced by multiple factors, no specific type of leadership can be considered as most appropriate. Furthermore, this review showed a high level of overlap between the behaviors of the many types of leadership presented in the articles included. It is, therefore, questionable whether leadership styles are a useful vocabulary in the debate on leadership in nursing homes. Moreover, the current tendency toward flat organizations, decentralization and self-direction transforms leadership into a more collective undertaking that transcends hierarchy and encompasses behavior, context and people. Tendencies toward networks of collaborating organizations require new leadership competencies that transcend organizational boundaries and interests. Therefore, a stronger focus on leadership behaviors in relationship to specific contexts instead of the application of leadership styles could provide more insight into what is needed when and what works.

The findings of this study show that leadership is a complex and multidimensional phenomenon, which is determined by multiple internal and external factors. Employees of nursing homes have to be aware that the success of leadership is determined by the interplay between behavior and several contextual factors and the various people involved. Furthermore, the study findings suggest focusing more on leadership behaviors instead of styles. Although thinking in leadership styles could be helpful in terms of categorization and framing, a broader and more conceptual perspective on leadership could be helpful in providing more insight into the underlying mechanisms and behaviors that play a role in leadership. First, a broader perspective implies that leadership should be seen as more than merely a function for managers and team leaders (Martin and Learmonth, 2012). It should be constructed as something to be enacted by all employees across an organization. Second, the broader perspective also means that one has to be aware that leadership processes take place at multiple layers in an organization, e.g. in the care setting, in professional interaction or at the board level. Third, people in organizations could benefit from more awareness of their leadership behavior and how this fits with the current context, circumstances and developments.

## Figures and Tables

**Table 1.** Characteristics of the articles included

Study type	Study design	*n*
Qualitative	Case study	5
	Descriptive	10
	Ethnography	2
Quantitative	Randomized controlled trial	1
	Non-randomized: a cross-sectional analytical study	9
	Descriptive	6
Mixed methods	Sequential explanatory design	1
	Embedded design	2
	Triangulation design	1
Review	Review	2
	Systematic review	5

**Table 2.** Leadership styles and their associated characteristics and behaviors

Leadership style	Behavioral characteristics	Reference
*Relationship-oriented leadership styles*		
Relational leadership	Focused on developing and maintaining relationships with staff, using emotional skills such as listening, empathy and responding to concerns (Cummings *et al.*, 2008)	Cummings *et al.* (2008, 2010), Forbes-Thompson *et al.* (2007), Havig *et al.* (2011), Havig *et al.* (2011), Jeon *et al.* (2015) and Kristiansen *et al.* (2016)
Transformational leadership	Facilitates change, strengthens the commitment of staff, increases job satisfaction and well-being within teams (Nielsen *et al.*, 2009)	Cummings *et al.* (2008, 2010), Hakanson *et al.* (2014), Jennings *et al.* (2011), Jeon *et al.* (2015), Jeon *et al.* (2015), Keisu *et al.* (2018), Marotta (2010), Nielsen *et al.* (2008, 2009, 2010), Nielsen and Cleal (2011), Nielsen and Munir (2009), Olinger (2010), Rokstad *et al.* (2015) and Westerberg and Tafvelin (2014)
*Individualized consideration*	*Not clearly described*	Cummings *et al.* (2010)
Resonant leadership	Inspires, coaches, develops and includes staff (Cummings *et al.*, 2010)	Cummings *et al.* (2010), Jennings *et al.* (2011) and Vesterinen *et al.* (2009)
Coaching (incl. managerial coaching)	Facilitating, empowering and increasing the confidence of staff, using competencies as listening, appreciation and involvement (Cummings *et al.*, 2014)	Buljac-Samardzic and van Woerkom (2015), Cummings *et al.* (2014) and Vesterinen *et al.* (2009)
Consensus manager leadership	Staff is asked for input and decision-making in teams is stimulated (Donoghue and Castle, 2009)	(Castle and Decker (2011), Donoghue and Castle (2009) and McKinney *et al.* (2016)
Consultative autocrat	Staff is asked for input, but eventually, the consultative autocrat takes the decision (Donoghue and Castle, 2009)	Castle and Decker (2011), Donoghue and Castle (2009) and McKinney *et al.* (2016)
*Authentic leadership*	*Not clearly described*	Hakanson *et al.* (2014)
Compassionate leadership	A compassionate leader leads with “head and heart,” recognizes and involves both cognitive and affective domains, behaving in a friendly, honest and consistent manner (Ali and Terry, 2017)	Ali and Terry (2017)
Appreciative management	Appreciative management is based on moral principles and the appreciation of human dignity (Astala *et al.*, 2017)	Astala *et al.* (2017)
*Connective leadership*	*Not clearly described*	Jennings *et al.* (2011)
Servant leadership	Based on trust, empowerment and development of teams. Based on collective needs instead of individual needs (Cloutier *et al.*, 2016)	Cloutier* et al.* (2016)
Active leadership	Shows active leadership at different hierarchical levels, takes decisions, no conflicts between leaders and staff. Promotes the realization of the mission (Quasdorf and Bartholomeyczik, 2019)	Havig and Hollister (2018) and Quasdorf and Bartholomeyczik (2019)
Participative leadership	Involves staff and give them a chance to grow to succeed in the process of changing the culture, highlights growth and creativity, views risk-taking as important for innovation (not rule-bound) (Brodtkorb *et al.*, 2019)	(Brodtkorb *et al.* (2019)
“Partnered” *leadership*	*Not clearly described*	Jennings *et al.* (2011) and Leutz *et al.* (2010)
Task-oriented leadership styles		
Task-oriented leadership	Highlights planning of tasks and activities (who does what, when and how), division of roles, goalsetting, monitoring and results (Havig et al., 2011)	Havig *et al.* (2011)
Transactional leadership	Based on transaction and exchange between leaders, colleagues and other people involved (Cummings *et al.*, 2010)	Cummings *et al.* (2010) and Jennings *et al.* (2011)
Autocratic leadership	Staff has a low level of autonomy. The autocrat does not ask staff for input and takes the decision individually (Donoghue and Castle, 2009)	Castle and Decker (2011), Donoghue and Castle (2009) and McKinney *et al.* (2016)
Shareholder management	Staff has a high level of autonomy, but the leader does not communicate about decision-making and expectations (Donoghue and Castle, 2009)	Castle and Decker (2011), Donoghue and Castle (2009) and McKinney *et al.* (2016)
Laissez-faire	Passive leadership, the minimal exchange between leaders and followers, abdication of authority and avoidance of decision-making (Quasdorf and Bartholomeyczik, 2019)	Cummings *et al.* (2010), Jennings *et al.* (2011) and Quasdorf and Bartholomeyczik (2019)
*Management by exception*	*Not clearly described*	Cummings *et al.* (2010)
*Dissonant leadership*	*Not clearly described*	Cummings *et al.* (2010)
*Instrumental leadership*	*Not clearly described*	Cummings *et al.* (2010)
*Non-resonant leadership*	*Not clearly described*	Cummings *et al.* (2010)
Context-dependent leadership styles		
Situational leadership	Assuming that there is no universal leadership style fitting in all contexts and situations (Lynch *et al.*, 2011)	Lynch *et al.* (2011, 2018) and Rokstad *et al.* (2015)
Adaptive leadership	Increasing people’s ability to cope with complex problems (Corazzini *et al.*, 2015; Corazzini and Anderson, 2013)	Corazzini *et al.* (2015) and Corazzini and Anderson (2013)

**Table 3.** Reported effects of leadership

Category	Positive effect	Application of	Reference
Effects of leadership on health-care employees	Positive effect on job satisfaction and low turn-over	Relational leadership styles	Cummings *et al.* (2010), Donoghue and Castle (2009), Havig *et al.* (2011) and Nielsen *et al.* (2009)
		Task-oriented leadership	Havig *et al.* (2011)
		“Strong and effective leadership”	Jeon *et al.* (2010)
	Positive effect on (for example) a higher organizational commitment	Relational leadership styles	Cummings *et al.* (2010), Donoghue and Castle (2009), Lundgren *et al.* (2016) and Nielsen *et al.* (2009)
		“Strong and effective leadership”	Jeon *et al.* (2010)
	Positive effect on health and well-being (including appreciation (equality)	Relational leadership styles	Astala *et al.* (2017), Cummings *et al.* (2010), Keisu *et al.* (2018), Nielsen *et al.* (2008, 2009) and Nielsen and Munir (2009)
	Positive effect on the work culture and the psychosocial climate	Relational leadership styles	Backman *et al.* (2016, 2018), Cummings *et al.* (2014, 2010), Lundgren *et al.* (2016) and Nielsen *et al.* (2008, 2010)
	Positive effect on productivity/effectiveness	Relational leadership styles	Buljac-Samardzic and van Woerkom (2015) and Cummings *et al.* (2010)
		“Strong and effective leadership”	Jeon *et al.* (2010)
	Positive effect on empowerment/growth and development opportunities	Relational leadership styles	Cummings *et al.* (2014, 2010), Lundgren *et al.* (2016) and Nielsen *et al.* (2008)
Effects of leadership on quality of care	Positive effect on the quality of care	Task-oriented leadership combined with relational leadership styles	Jennings *et al.* (2011)
		Relational leadership styles	Castle and Decker (2011), Harvath *et al.* (2008), McKinney *et al.* (2016) and Westerberg and Tafvelin (2014)
		Task-oriented leadership	Havig *et al.* (2011)
		“Strong and effective leadership”	Jeon *et al.* (2010)
		Studied, but no effects reported	Jeon *et al.* (2015), Marotta (2010), Olinger (2010) and Westerberg and Tafvelin (2014)
Effects of leadership on quality of life	Positive effect on the quality of life	Relational leadership styles	McKinney *et al.* (2016)
Effects of leadership on person-centered care	Positive effect on person-centeredness of care	Leadership behavior in general	Backman *et al.* (2016), Brodtkorb *et al.* (2019) and Lynch *et al.* (2011)
Effects on innovation processes	Positive effect on innovation processes	Relation leadership styles	Brodtkorb *et al.* (2019)

**Table 4.** Factors influencing leadership

Category	Factor	Reference
The leader	Personal characteristics	Cummings *et al.* (2008, 2014) and Nielsen and Cleal (2011)
	Leadership competencies	Cummings *et al.* (2008)
	Educational activities of the leader	Cummings *et al.* (2008), Hakanson *et al.* (2014) and Vesterinen *et al.* (2009)
	Distance to practice	Havig and Hollister (2018) and Kristiansen *et al.* (2016)
The team(s)	Turnover and absence	Cloutier *et al.* (2016) and Havig *et al.* (2011)
	Interpersonal relations	Corazzini *et al.* (2015) and Havig and Hollister (2018)
	Workload	Corazzini *et al.* (2015) and Westerberg and Tafvelin (2014)
	Willingness to be coached	Cummings *et al.* (2014) and Havig *et al.* (2011)
	Employee well-being and satisfaction	Cummings *et al.* (2014) and Nielsen *et al.* (2008)
	Self-efficacy	Nielsen *et al.* (2009) and Nielsen and Munir (2009)
	Interdependent workgroups	Havig and Hollister (2018)
The organization	Organizational structure	Corazzini *et al.* (2015), Cummings *et al.* (2008, 2014), Hakanson *et al.* (2014), Lundgren *et al.* (2016), Lynch *et al.* (2011) and Rokstad *et al.* (2015)
	Implementation of person-centered care	Backman *et al.* (2016, 2020)
	Organizational culture	Ali and Terry (2017), Backman *et al.* (2020), Corazzini *et al.* (2015), Havig and Hollister (2018), Jeon *et al.* (2010), Nielsen *et al.* (2008) and Vesterinen *et al.* (2009)
	Available information and information flow	Forbes-Thompson *et al.* 2007), Hakanson *et al.* 2014), Jeon *et al.* (2010) and Vesterinen *et al.* (2009)
	Earlier superiors	Vesterinen *et al.* (2009)
	Available budget and time	Ali and Terry (2017), Cummings *et al.* (2014), Hakanson *et al.* (2014), Nielsen *et al.* (2010) and Rokstad *et al.* (2015)
	Tasks and responsibilities	Hakanson *et al.* (2014), Jeon *et al.* (2010), Kristiansen *et al.* (2016) and Nielsen *et al.* (2008)
	Leadership team	Hakanson *et al.* (2014) and Vesterinen *et al.* (2009)
	Organizational dynamics and stability	Jeon *et al.* (2010), Nielsen *et al.* (2010) and Nielsen and Cleal (2011)
	Support from superiors	Jeon *et al.* (2010) and Westerberg and Tafvelin (2014)
	Openness to change and innovation	Brodtkorb *et al.* (2019), Jeon *et al.* (2010), Lynch *et al.* (2011) and Nielsen *et al.* (2008)
